# A High Molar Extinction Coefficient Bisterpyridyl Homoleptic Ru(II) Complex with *trans*-2-Methyl-2-butenoic Acid Functionality: Potential Dye for Dye-Sensitized Solar Cells

**DOI:** 10.3390/ijms13033511

**Published:** 2012-03-14

**Authors:** Adewale O. Adeloye, Temitope O. Olomola, Akinbulu I. Adebayo, Peter A. Ajibade

**Affiliations:** 1 Department of Chemistry, Faculty of Science and Agriculture, University of Fort Hare, P.M.B. X1314, Alice 5700, South Africa; E-Mail: pajibade@ufh.ac.za; 2 Department of Chemistry, Faculty of Science, Rhodes University, P.M.B. 94, Grahamstown 6140, South Africa; E-Mail: oloms2002@yahoo.co.uk; 3 Department of Chemistry, Faculty of Science, University of Lagos, Akoka, Lagos State, Nigeria; E-Mail: iakinbulu@unilag.edu.ng

**Keywords:** homoleptic Ru(II) complex, terpyridine, 2-Methyl-2-butenoic acid, extended-π-bond conjugation, spectroscopy, molar extinction coefficient, electrochemistry

## Abstract

In our continued efforts in the synthesis of ruthenium(II) polypyridine complexes as potential dyes for use in varied applications, such as the dye-sensitized solar cells (DSSCs), this work particularly describes the synthesis, absorption spectrum, redox behavior and luminescence properties of a new homoleptic ruthenium(II) complex bearing a simple *trans*-2-methyl-2-butenoic acid functionality as the anchoring ligand on terpyridine moiety. The functionalized terpyridine ligand: 4′-(*trans*-2-methyl-2-butenoic acid)-terpyridyl (L1) was synthesized by aryl bromide substitution on terpyridine in a basic reaction condition under palladium carbide catalysis. In particular, the photophysical and redox properties of the complex formulated as: bis-4′-(*trans*-2-methyl-2-butenoic acid)-terpyridyl ruthenium(II) bis-hexafluorophosphate [Ru(L1)_2_(PF_6_)_2_] are significantly better compared to those of [Ru(tpy)_2_]^2+^ and compare well with those of the best emitters of Ru(II) polypyridine family containing tridentate ligands. Reasons for the improved photophysical and redox properties of the complex may be attributed partly to the presence of a substituted α,β-unsaturated carboxylic acid moiety leading to increase in the length of π-conjugation bond thereby enhancing the MLCT-MC (Metal-to-ligand-charge transfer-metal centred) energy gap, and to the reduced difference between the minima of the excited and ground states potential energy surfaces.

## 1. Introduction

The search for new energy systems based on renewable sources which require less energy consumption and are more environmentally friendly draws more and more attention. Over the past years, significant emphasis has been put on the development and understanding of light-driven charge separation in molecular systems as methods of converting and storing solar energy. Dye-sensitized solar cells (DSSCs) are one of the most promising new generation systems [1,2] due to their reasonably light energy conversion efficiency, low material and production costs and from the viewpoint of the life cycle assessment in mass production. In recent years, many groups have focused their attention on fundamental aspects of dye-sensitized solar cell components [3–10]. Dye-derivatized mesoporous titania film is one of the key components in such cells. The electrochemical, photophysical, and ground- and excited-state properties of the dye play an important role for charge-transfer dynamics at the semiconductor interface [11]. The electron injection rates have been measured in different laboratories using the *cis-*di(thiocyanato)-bis-(2,2′-bipyridyl-4,4′-dicarboxylate) ruthenium(II) complex (referred as N3) and were found to occur in the femtosecond time scale [12].

The optimal sensitizer for the dye-sensitized solar cell should be panchromatic, that is, absorb visible light of all colors. Ideally, all photons below a threshold wavelength of about 920 nm should be harvested and converted to electric current. This limit is derived from thermodynamic considerations showing that the conversion efficiency of any single-junction photovoltaic solar converter peaks at approximately 33% near threshold energy of 1.4 eV [13]. In addition, the sensitizer should fulfill several demanding conditions: (i) it must be firmly grafted to the semiconductor oxide surface and inject electrons into the conduction band with a quantum yield of unity; (ii) its redox potential should be sufficiently high so that it can be regenerated rapidly via electron donation from the electrolyte or a hole conductor; (iii) it should be stable enough to sustain at least 10^8^ redox turnovers under illumination corresponding to about 20 years of exposure to natural sunlight [14].

Ruthenium(II) polypyridine complexes have played important roles in several areas of research connected with solar energy conversion over the last three decades [15]. The prototype of this class of compound, [Ru(bpy)_3_]^2+^ (bpy ) 2,2′-bipyridine), is one of the most studied metal-containing species due to a combination of high photostability and interesting electrochemical and photophysical properties [16]. The coordination of the polypyridyl ligand 2,2′-6′,2″-terpyridine to ruthenium (II) affords very stable complexes and has attracted attention in recent years [17–19]. These complexes as inorganic dyes possess highly versatile luminescent and photoredox properties useful for the development of photochemistry, photophysics, photocatalysis, photoelectrochemistry, electron and energy transfer processes [20].

The spectral and redox properties of ruthenium polypyridyl complexes can be tuned in two ways. First, by introducing a ligand with a low-lying π* molecular orbital and, secondly, by destabilization of the metal t_2_g orbital through the introduction of a strong donor ligand. Meyer *et al.* have used these strategies extensively to tune the MLCT transitions in ruthenium complexes [21]. Heteroleptic complexes containing bidentate ligands with low-lying π* orbitals, together with others having strong σ-donating properties, express impressive panchromatic absorption properties [22]. However, the extension of the spectral response into the near-IR was gained at the expense of shifting the LUMO orbital to lower levels from where charge injection into the TiO_2_ conduction band can no longer occur [23]. Near-infrared response can also be gained by upward shifting of the Ru t_2_g (HOMO) levels. However, it turns out that the mere introduction of strong sigma donor ligands into the complex often does not lead to the desired spectral result as both the HOMO and LUMO are displaced in the same direction. Furthermore, the HOMO position cannot be varied freely as the redox potential of the dye must be maintained sufficiently positive to ascertain rapid regeneration of the dye by electron donation from iodide, following charge injection into the TiO_2_ [24].

In our continued efforts to contribute to the demanding requirements of the sensitizers for the dye-sensitized solar cells, we have engineered, at the molecular level, and synthesized a ruthenium(II) complex in which the ruthenium center is coordinated to a simple *trans*-2-methyl-2-butenoic acid-terpyridyl at the 4′-position. The purpose of incorporating an α,β-unsaturated carboxylate group in the ligand is three-fold: (i) to increase the molar extinction coefficient of the complex through π-bond elongation; (ii) to facilitate the grafting of the dye on the semiconductor surface; and (iii) to ensure intimate electronic coupling between its excited-state wave function and the conduction band manifold of the semiconductor. It is well-known that substitution of carboxy groups at the 4,4′-positions of 2,2′-bipyridine affords an increase in the molar extinction coefficient of 40% [25]. The role of the *trans* methyl groups as substituents on the acid ligand is to possibly tune, enhance stability and/or contribute to the electron donating ability of the complex. In comparison to other synthesized ruthenium(II) bipyridyl and phenanthrolyl complexes earlier published [26–28], this paper reports most essentially an improved molar extinction coefficient, luminescence and redox properties of a new homoleptic bis-4′-(*trans*-2-methyl-2-butenoic acid)-terpyridyl ruthenium(II) bis-hexafluorophosphate [Ru(L1)_2_(PF_6_)_2_] complex.

## 2. Results and Discussion

### 2.1. Synthesis

**Scheme 1:**
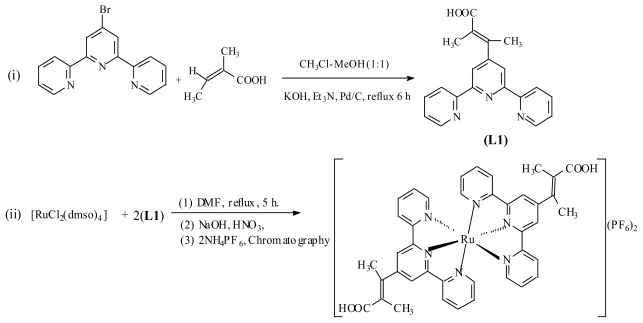
Synthetic pathways for Ligand L1 and [Ru(L1)_2_](PF_6_)_2_ complex.

The reaction of 4′-bromoterpyridine and *trans*-2-Methyl-2-butenoic acid proceeded under a catalyzed palladium cross-coupling reaction in a basic medium and using high temperature afforded the desired coupling product as 4′-(*trans*-2-Methyl-2-butenoic acid)-terpyridine (L1) and the corresponding homoleptic complex [Ru(L1)_2_(PF_6_)_2_] resulted from its reaction with [RuCl_2_(dmso)_4_]. The complex was purified using Sephadex LH-20 adsorbent and diethyl ether-methanol solvent mixture as mobile phase. The complex was precipitated using a 0.50 g of aqueous solution of ammonium hexafluorophosphate (Scheme 1).

### 2.2. Infrared Spectra Studies of the Ligand and Complex

**Figure 1:**
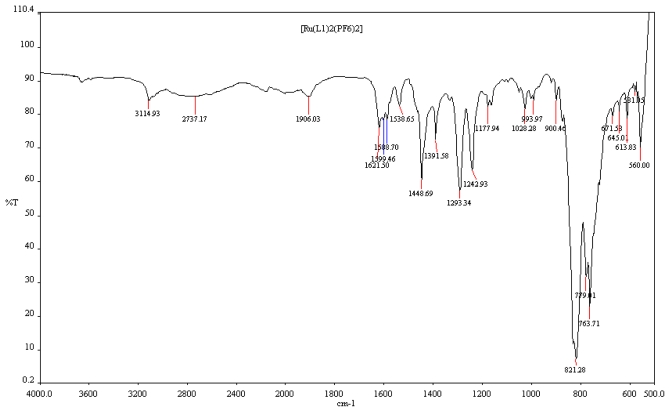
FT-IR spectrum of [Ru(L1)_2_(PF_6_)_2_] complex.

The infrared spectra of the ligand L1 and [Ru(L1)_2_(PF_6_)_2_] complex share common features, one of which is the strong broad bands in the region of 2737 cm^−1^ and 3114 cm^−1^ due to hydroxyl groups of the carboxylic acid moieties on the complexes. The spectrum of the complex (Figure 1) displays a broad asymmetric carboxylate stretching bands ν_asym_(COO^−^) in the region 1906 and 1621 cm^−1^. Both bands are indicative of a protonated carboxylate group on the terpyridine, if the ligand was partially deprotonated, ν_asym_(COO^−^) would be expected at ~1600 cm^−1^ [26]. However, C=C and C=N stretching bands of the terpyridyl ligands also appear near 1600 cm^−1^ and of reduced intensity when compared to the uncoordinated ligand, making it difficult to determine if the H-tpy ligand is fully or only partially protonated on this basis. Aside from the small variations in the ν_asym_(COO^−^) bands and a band at 1392 cm^−1^ in [Ru(L1)_2_(PF_6_)_2_], the IR spectrum of the complex is in good agreement. At the fingerprint regions, the strong absorption frequencies at 821 and 763 cm^−1^ are associated with the hexafluorophophate counter-ion present in the molecule while the weak absorption band frequencies at 671–613 cm^−1^ show the mono-substitution pattern of the anchoring *trans*-2-methyl-2-butenoic acid on the terpyridyl ligand [29–31].

### 2.3. ^1^H and ^13^C-NMR Spectroscopic Studies

**Figure 2:**
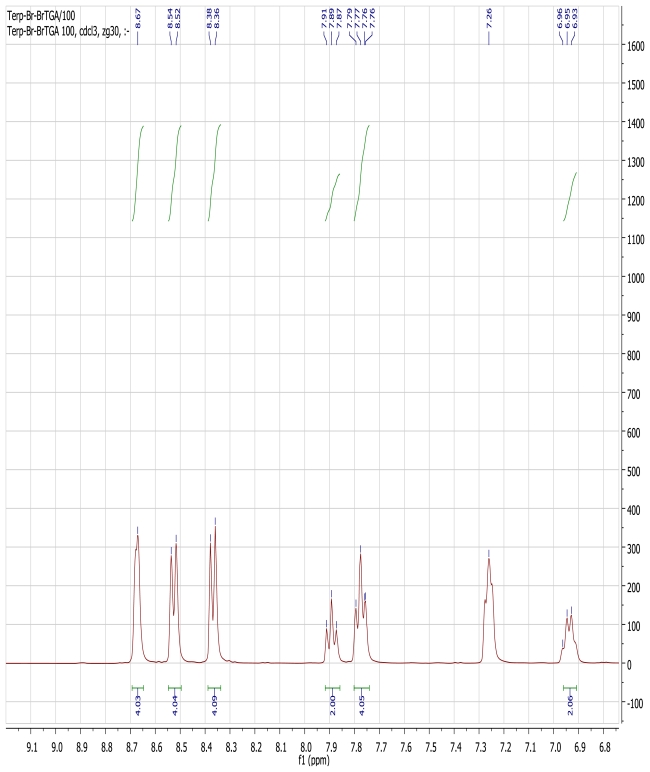
Aromatic region of ^1^H-NMR spectrum of [Ru(L1)_2_](PF_6_)_2_ complex in CDCl_3_.

**Figure 3:**
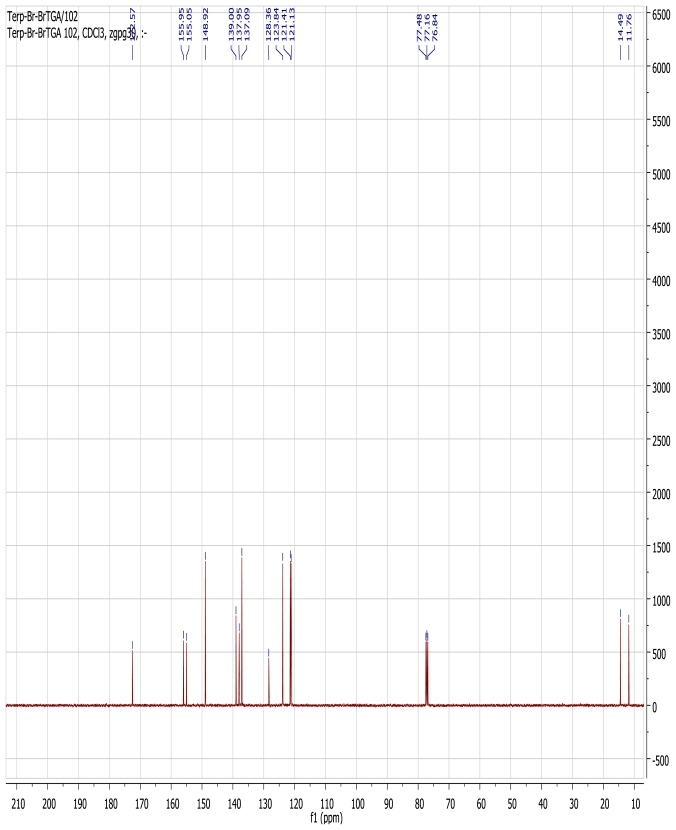
^13^C-NMR spectrum of [Ru(L1)_2_](PF_6_)_2_ complex in CDCl_3_.

The ^1^H NMR spectrum of the ligand L1, measured in CDCl_3_ solution shows five sharp and well resolved signals in the aromatic region, corresponding to the terpyridyl protons in which the two peripheral rings are magnetically equivalent. A broad aromatic singlet signal at δ 8.63 ppm is assigned to H-3′ and H-5′. Two doublets resonance signals at 8.55 and 8.38 (d, *J* = 8.0 Hz, 2H) were respectively assigned to H-6, 6″ and H-3, 3″, while the triplet signals at δ 7.89 and 7.79 ppm (t, *J* = 8.0 Hz, 2H) were assigned to the H-5, 5″, and H-4, 4″ respectively. At the aliphatic region, a singlet peak at 2.09 ppm was unambiguously assigned to the CH_3_ signal. In a pseudo-octahedral geometry, a tridentate ligand like terpyridine coordinates to a metal center in a meridional fashion [32] thus, in the proton NMR spectrum of the homoleptic [Ru(L1)_2_(PF_6_)_2_] complex in CDCl_3_, a slight complication in the aromatic region was observed. The complication may be due to a different geometrical arrangement occasioned by the two outer pyridine rings of the terpyridine and/or the coordination of the nitrogen atom lone-pair electrons to the ruthenium ion centre leading to an upfield shifts in the chemical shifts of protons (Figure 2). The proton spectrum showed signals between δ 8.68–6.94 ppm integrating for twenty protons expected from the two coordinating ligands. The aliphatic region of the spectrum, however, showed the two signals at δ 1.81 and 1.74 ppm that were integrated for twelve protons of the *trans-*methyl signals of the anchoring ligand. The ^13^C-NMR spectrum data of the complex, however, showed the expected carbon atom signals of both the terpyridine and the 2-methyl-2-butenoic acid moieties (Figure 3). The carbonyl signal at δ 172.52 ppm was unambiguously assigned to the carboxylic acid signal, and the reduced intensity signals at δ 155.95, 155.05, 139.00, 137.95 and 128.36 ppm were assigned to the five quaternary carbon atoms in the molecule. Five strong signals at δ 148.92, 137.09, 123.84, 121.41 and 121.13 ppm were of the terpyridyl carbons and the two methyl signals from the *trans* 2-methyl-2-butenoic acid moiety were found at 14.49 and 11.76 ppm respectively.

### 2.4. Electronic Absorption Spectroscopic Study of the [Ru(L1)_2_(PF_6_)_2_] Complex

**Figure 4:**
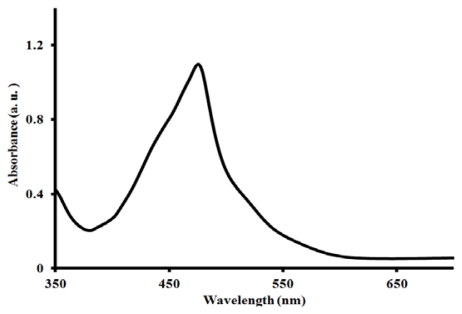
UV-Vis absorption spectrum of complex [Ru(L1)_2_(PF_6_)_2_] in acetonitrile.

Shown in Figure 4, the UV-Vis absorption spectrum of [Ru(L1)_2_(PF_6_)_2_] complex in acetonitrile. The complex displays intense band in the ultraviolet region between 200–300 nm (not shown), this was assigned to the intra ligand π→π* charge-transfer transition of the terpyridine ligand. The complex exhibits a very broad and intense metal-to-ligand charge (MLCT) absorption band throughout the visible region of the spectrum (400–600 nm), characteristic of many other ruthenium(II) polypyridyl complexes, and which can be assigned to electronic transitions from the Ru^II^ based t_2_g orbital to the ligand based π* orbitals. The molar extinction coefficient of complex [Ru(L1)_2_(PF_6_)_2_] at its maximum (479 nm) is 10 400 M^−1^·cm^−1^. The lower-energy absorption in the complex was enhanced due to the presence of the electron withdrawing nature of the carboxylic groups, which lowers the energy of the π* orbital of the terpyridine ligand, an electron-donating group provided by the *trans* nature of the methyl groups destabilizes the reduction process and thus, expected to further improve the stability of the complex. The metal complex so obtained exhibits appreciable photophysical properties, most especially its molar extinction coefficient in the visible region which is significantly better compared to some that have already been reported for [Ru(tpy)_2_]^2+^ [33–36]. However, the complex exhibits a lower wavelength characteristic compared to the N3 and N719 standard dyes [14,31].

### 2.5. Emission Spectroscopic Study of the [Ru(L1)_2_(PF_6_)_2_] Complex

**Figure 5:**
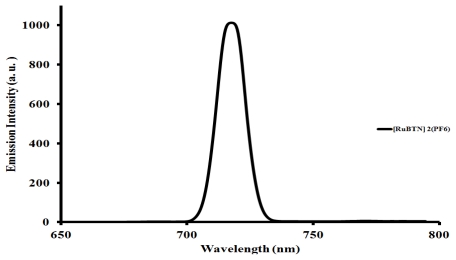
Emission spectrum of complex [Ru(L1)_2_(PF_6_)_2_] in acetonitrile.

The emission spectrum of complex [Ru(L1)_2_(PF_6_)_2_] is displayed in Figure 5. Upon excitation into the ^1^LC and ^1^MLCT bands, (*λ*_exc._ = 470 nm), the complex displays appreciable luminescence at room temperature. An emission wavelength maximum was found at 715 nm. It is well known that conjugated functional organic molecules are useful for the study of electron transport at the molecular scale and that the use of fused-ring systems is a powerful and practical approach. The luminescent properties of a complex as well as its ability to play the role of excited state reactant or product are related to the energy ordering of its low energy excited state and, particularly, to the orbital nature of its lowest excited state. It has been shown that the stronger the electron withdrawing group at the 4′-position of the terpyridine, the stronger the luminescence [8,9,15]. Thus, the choice of ligands has significant effect, which in turn influences the energy positions of the metal centre (MC) and ligand centre (LC), as well as the metal-to-ligand transfer (MLCT) of ruthenium(II) polypyridine complexes [20]. The energy of the MC excited state depends on the ligand field strength, which in turn depends on the σ-donor and π-acceptor properties of the ligands, the steric crowding around the metal (that can preclude a sufficiently close approach between metal and ligand), and the bite angle of the polydentate ligands (which in some cases cannot be optimized because of molecular constraint). The energy of the LC excited state depends on the intrinsic properties of the ligands, such as the HOMO-LUMO energy gap and the singlet-triplet splitting. It has been shown that the energy of the MLCT excited state depends on the reduction potential of the ligand involved in the MLCT transition, the oxidation potential of the metal in the complex (which is affected by the electron donor and acceptor properties of the ligands), and by the charge separation caused by the transition [37–44]. In the complex [Ru(L1)_2_(PF_6_)_2_], the intense emission is a significant contribution to the excited state from an interaction between the metal *d*-orbital and the ligand π-systems [28].

### 2.6. Electrochemical Study

**Figure 6:**
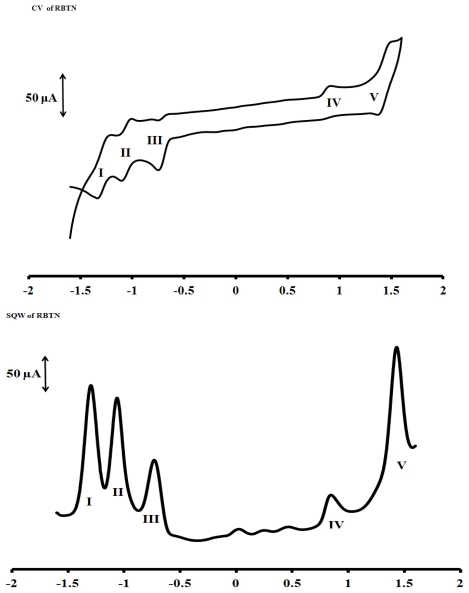
Cyclic voltammetric and Square wave for [Ru(L1)_2_(PF_6_)_2_] complex at 1 × 10^−3^ M in freshly distilled DMF containing 0.1 M TBABF_4_ supporting electrolyte. Step potential = 5 mV, amplitude = 50 mV *vs.* Ag|AgCl, frequency = 10 Hz. Scan rate = 100 mVs^−1^ *vs.* Ag|AgCl.

The cyclic and square wave voltammograms of the complex [Ru(L1)_2_(PF_6_)_2_] were examined in the potential range +1.5 to −1.5 V and at a scan rate 50 mV·s^−1^ using Ag|AgCl electrode in DMF solvent with 0.1 M tetrabutylammonium hexafluorophosphate as supporting electrolyte (Figure 6). The voltammograms display the Ru(III)/Ru(II) couple at positive potential and ligand based reduction couples at negative potentials. A reversible one-electron oxidation process **V** at E_1/2_ = +1.42 V was assigned to the metal centre Ru(III)/Ru(II) wave couple [17]. One unidentified irreversible oxidation peak process **IV** was observed at +0.82 V, which was tentatively ascribed to the ring oxidation of the terpyridine ligand and, as well as the carboxylate ions present in the complex. At the negative potential, three reversible waves were observed at −1.31, −1.07, and −0.75 V for processes **I**, **II** and **III** respectively. The more positive reduction potential may be assigned to the contribution from the ligand due to an additional (C=C) conjugative π-bond as found in the structure of terpyridine since the ligand contains the electron withdrawing COOH group of the anchoring ligand being responsible for lowering the LUMO levels [26].

## 3. Experimental Section

All chemical and reagents were analytically pure and used without further purification. 4′-Bromo-2,2′:6′,2″-terpyridine was synthesized as described in the literature [45]. 4′-(*trans*-2-Methyl-2-butenoic acid)-2,2′:6′,2″-terpyridine (L1) was synthesized following the literature procedure [12] with slight modifications (Scheme 1). All thin layer chromatography (TLC) analyses were done on aluminium sheets precoated with normal phase silica gel 60 F_254_ (Merck, 0.20 mm thickness), unless otherwise stated. The TLC plates were developed using any of the following solvent systems: Solvent system A: dichloromethane-methanol (9:1); Solvent system B: dichloromethane-methanol (7:3); Solvent system C: diethyl ether-methanol (1:1). Gel filtration was performed using Sephadex LH-20 previously swollen in specified solvent (s) prior to loading of extract onto the column (3.5 cm × 8.5 cm).

Melting points were determined using a Gallenkamp electrothermal melting point apparatus. Microanalyses (C, H, N) were carried out with a Fisons elemental analyzer and infrared spectra were obtained with KBr discs on a Perkin Elmer System 2000 FT-IR spectrophotometer. UV-Vis and fluorescence spectra were recorded in a 1 cm path length quartz cell on a Perkin Elmer Lambda 35 spectrophotometer and Perkin Elmer Lambda 45 spectrofluorimeter, respectively. ^1^H- and ^13^C-NMR spectra were run on a Bruker EMX spectrometer operating at 400 MHz for ^1^H and 100 MHz for ^13^C. The chemical shift values were reported in parts per million (ppm) relative to (TMS) as internal standard. Chemical shifts were reported for the ligands and complex with respect to CDCl_3_ at *δ*_c_ 77.00, *δ*_H_ CDCl_3_ at 7.26. Electrochemical experiment was performed using a PGSTAT 302 Autolab potentiostat (EcoChemie, Utrecht, The Netherlands) driven by the general purpose Electrochemical System data processing software (GPES, software version 4.9). A conventional three-electrode system was used. The working electrode was a bare glassy carbon electrode (GCE), Ag|AgCl wire and platinum wire were used as the pseudo reference and auxiliary electrodes, respectively. The potential response of the Ag|AgCl pseudo-reference electrode was less than the Ag|AgCl (3 M KCl) by 0.015 ± 0.003 V. Prior to use, the electrode surface was polished with alumina on a Buehler felt pad and rinsed with excess millipore water. All electrochemical experiments were performed in freshly distilled dry DMF containing TBABF_4_ as supporting electrolyte.

### 3.1. Synthesis of 4′-(*trans*-2-Methyl-2-butenoic Acid)-2,2′:6′,2″-Terpyridine (L1)

In a 100 mL flask, 4′-Bromo-terpyridine (0.42 g, 1.35 mmol) and *trans*-2-Methyl-2-butenoic acid (0.14 g, 1.35 mmol) were dissolved in chloroform/methanol (50 mL, 1:1, v/v). Triethylamine (1.0 mL) and palladium-carbide (0.050 g, 0.42 mmol) were added and the mixture put into reflux for 6 h at a temperature between 110–120 °C. The reaction was allowed to cool to room temperature and the solvent removed under reduced pressure. The pink residue was dissolved in degassed water and extracted with chloroform. The chloroform extract was concentrated *in vacuo* to afford purple solid which was recrystallized in diethyl-ether–ethanol (1:1, 30 mL, v/v) to afford compound (L1). Colour: Purple crystalline solid; Melting point: 65–67 °C; Yield: 0.41 g, 73 %. IR (KBr) *ν*_max_/cm^−1^: 3473, 3415, 3051, 1618, 1582, 1564, 1455, 1422, 1264, 1146, 1100, 1080, 1038, 989, 833, 761, 735, 622, 509, 473. ^1^H NMR (400 MHz, CDCl_3_): δ 8.63 (s, H-3′, H-5′, 2H), 8.55 (d, *J* = 8.0 Hz, H-6,6″, 2H), 8.38 (d, *J* = 8.0 Hz, H-3,3″, 2H,), 7.89 (t, *J* = 8.0 Hz, H-5,5″, 2H), 7.79 (t, *J* = 8.0 Hz, H-4,4″, 2H,), 2.09 (s, CH_3_). Elemental Analysis (%): Found: C, 72.15; H, 4.83; N, 12.33 C_20_H_17_N_3_O_2_ requires C, 72.49; H, 5.17; N, 12.68.

### 3.2. Synthesis of Bis-4′-(*trans*-2-Methyl-2-butenoic Acid)-2,2′:6′,2″-Terpyridyl Ruthenium(II) Bis-Hexafluorophosphate Complex [Ru(L1)_2_(PF_6_)_2_]

[RuCl_2_(dmso)_4_], used as metal precursor, was synthesized as reported [46]. In a 250 mL flask, 4′-(*trans*-2-methyl-2-butenoic acid)-2,2′:6′,2″-terpyridine (L1) (0.20 g, 0.61 mmol) and [RuCl_2_(dmso)_4_] (0.15 g, 0.30 mmol) were dissolved in dimethylformamide (40 mL) and refluxed for 5 h [47]. The crude product was concentrated *in vacuo* to afford red oil. To the crude product, 0.05 M NaOH solution was added to afford red-brown precipitate. The pH was adjusted to 3 with 0.5 M HNO_3_ which dissolves the precipitate. The solution was left to stand in the fridge (−2 °C) for 12 h before being filtered and concentrated *in vacuo*. The red oil solution gave rusty reddish-brown precipitate on addition of aqueous solution of ammonium hexafluorophosphate (0.5 g, 5 mL). The precipitate was filtered, washed in small amount of diethyl-ether and air-dried. Further purification of the crude solid product was achieved by column chromatography using Sephadex LH-20 adsorbent and solvent system C: (Diethyl ether-Methanol (1:1, v/v, 250 mL) to afford the desired product after removal of the solvent under reduced pressure and the oily product precipitated by aqueous ammonium hexafluorophosphate (0.5 g, 5 mL). Colour: Red-brown solid; Melting point: 210–213 °C; Yield: 0.12 g, 34%. IR (KBr) ν_cm-1_: 3114, 2737, 1906, 1621, 1599, 1540, 1530, 1440, 1391, 1293, 1242, 1171, 1020, 993, 900, 821, 779, 763, 671, 645, 613, 560. UV-Vis, CH_3_CN: *λ*_max_ = 479 nm (*ɛ* = 10 400 M^−1^·cm^−1^). Emission: *λ*_exc_ = 470 nm; *λ*_em_ = 715 nm. ^1^H- NMR (CDCl_3_): δ 10.27 (s, br, OH), 8.67 (s, 4H, H-3′, H-5′), 8.53 (d, *J* = 8.0 Hz, H-3,3″, 4H), 8.37 (d, *J* = 8.0 Hz, H-6,6″, 4H), 7.89 (t, 2H, H-5), 7.77 (t, 4H, H-4,4″), 6.95 (td, *J* = 8.0 Hz, 2H, H-5″), 1.81 (s, 6H, 2-CH_3_), 1.74 (d, 6H, 2-CH_3_). ^13^C-NMR (CDCl_3_): δ 172.57, 155.95, 155.05, 148.92, 139.00, 137.95, 137.09, 128.36, 123.84, 121.41, 121.13, 14.49 and 11.76. Electrochemical data: *E*_anodic_/V = 0.82, Ru^2+^/Ru^3+^ = 1.42 V, *E*_1/2_/V = −0.75, −1.07, −1.31 V. Elemental Analysis (%): Found: C, 45.28 H, 3.20; N, 7.49, RuC_40_H_34_N_6_O_4_P_2_F_12_ requires C, 45.59; H, 3.25; N, 7.98.

## 4. Conclusions

In conclusion, we have synthesized a new α,β-unsaturated carboxylic acid substituted terpyridine ligand and used it to prepare a homoleptic Ru(II) polypyridine complex. The metal complex so obtained exhibits photophysical and redox properties which are significantly better compared to some that have already been reported for [Ru(tpy)_2_]^2+^ and are also comparable with the best emitters of Ru(II) polypyridine complexes containing tridentate ligands. Reasons for the improved photophysical and redox properties relate to the presence of the electron withdrawing nature of the carboxylic groups which lowers the energy of the π* orbital. Hence, there is a smaller energy gap between the dπ orbitals of the metal centre and the π* orbital of the ligand; whereas, the ligand also contains two *trans-*methyl groups as electron-donating substituents that can stabilize the hole at the metal centre and increase the energy gap, which will increase quantum yield and lifetime. Thus, a push and pull characteristic is expected in the complex molecule. Because of the improved photophysical and redox properties, the complex investigated holds promise to be a suitable component for the dye-sensitized solar cells, larger supramolecular (multicomponent) systems based on Ru(II) tridentate polypyridine compounds capable of performing long-range photoinduced electron, therapeutic applications and/or energy transfer functions.

## Supplementary Information
