# Supplementary material for: A High Molar Extinction Coefficient Bisterpyridyl Homoleptic Ru(II) Complex with trans-2-Methyl-2-butenoic Acid Functionality: Potential Dye for Dye-Sensitized Solar Cells
Source: Int J Mol Sci. 2012 Mar 14;13(3):3511–26. doi: 10.3390/ijms13033511 (PMC3317725; doi:10.3390/ijms13033511)
Supplement: Supplementary file 1 [file ijms-13-03511-s001.pdf]

Supplementary Information

Supplementary 1. Structure and FT-IR spectrum of Ligand L1.

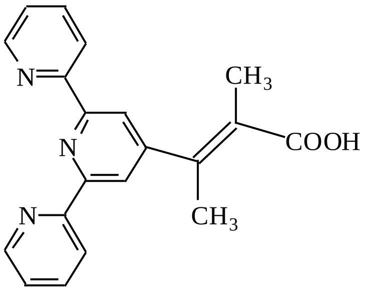

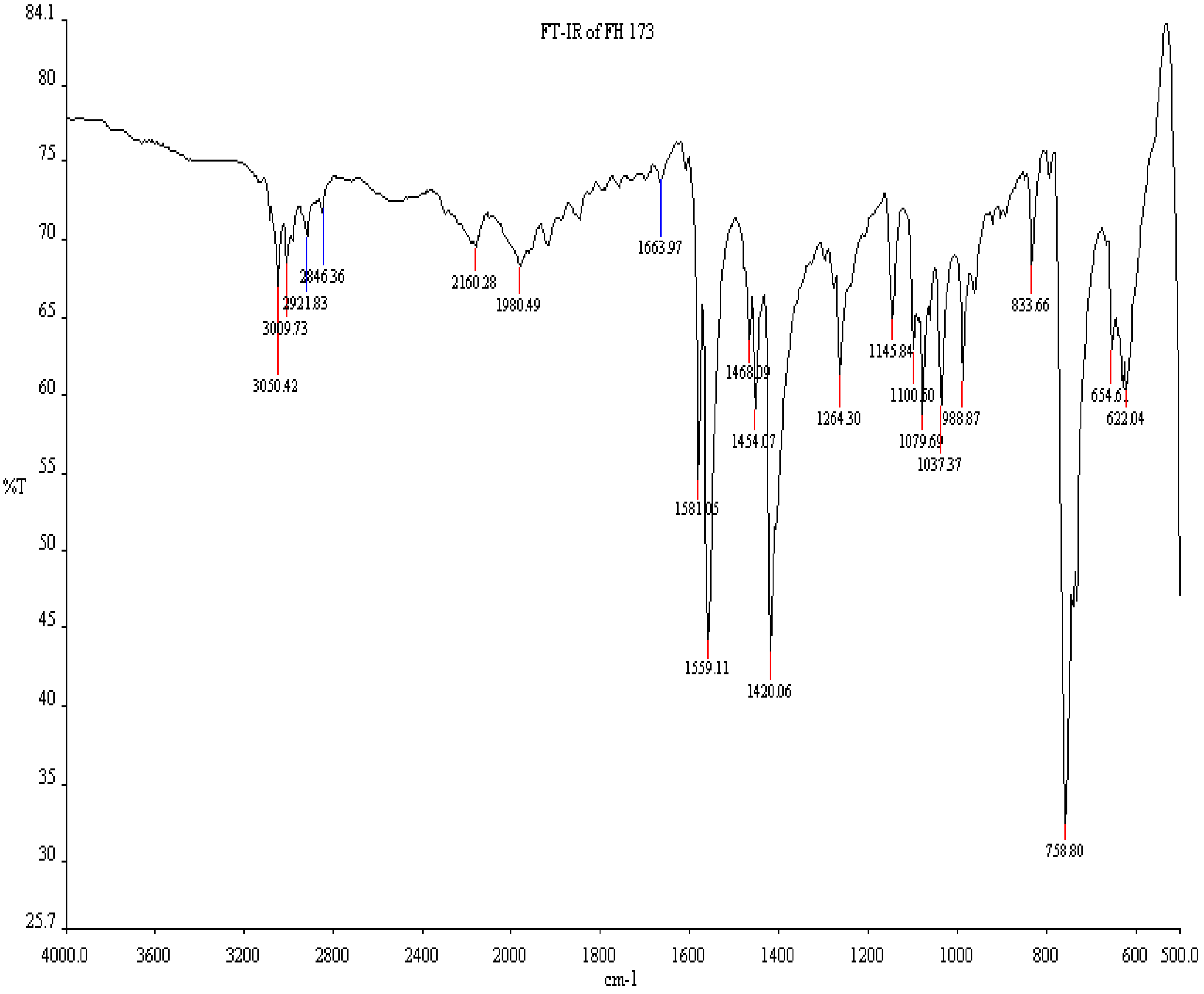

Supplementary 2. Structure and FT-IR spectra of unsubstituted terpyridine (red); compared to 4'-bromoterpyridine (black).

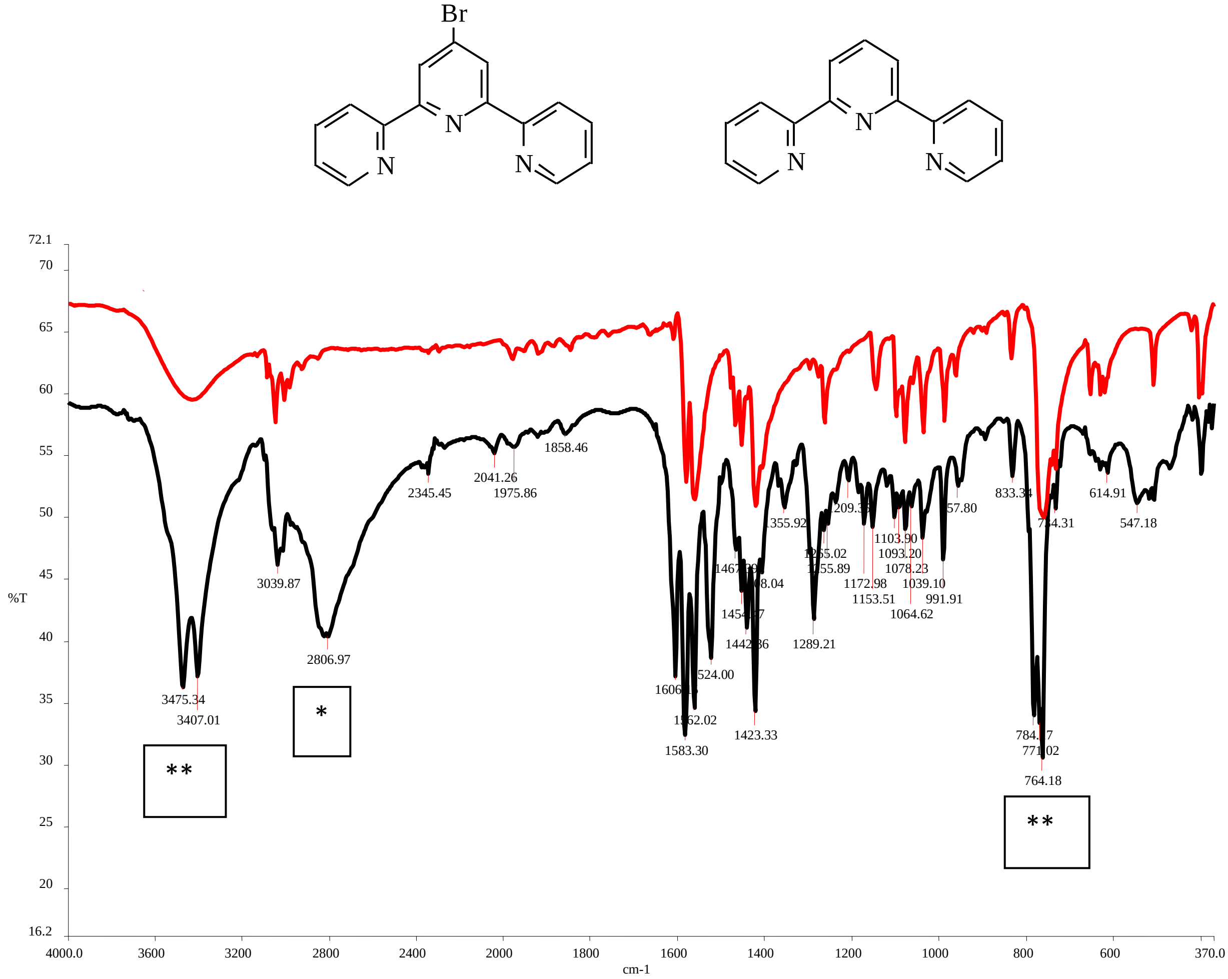

Supplementary 3. Aromatic region of 1H NMR spectrum of Ligand L1.

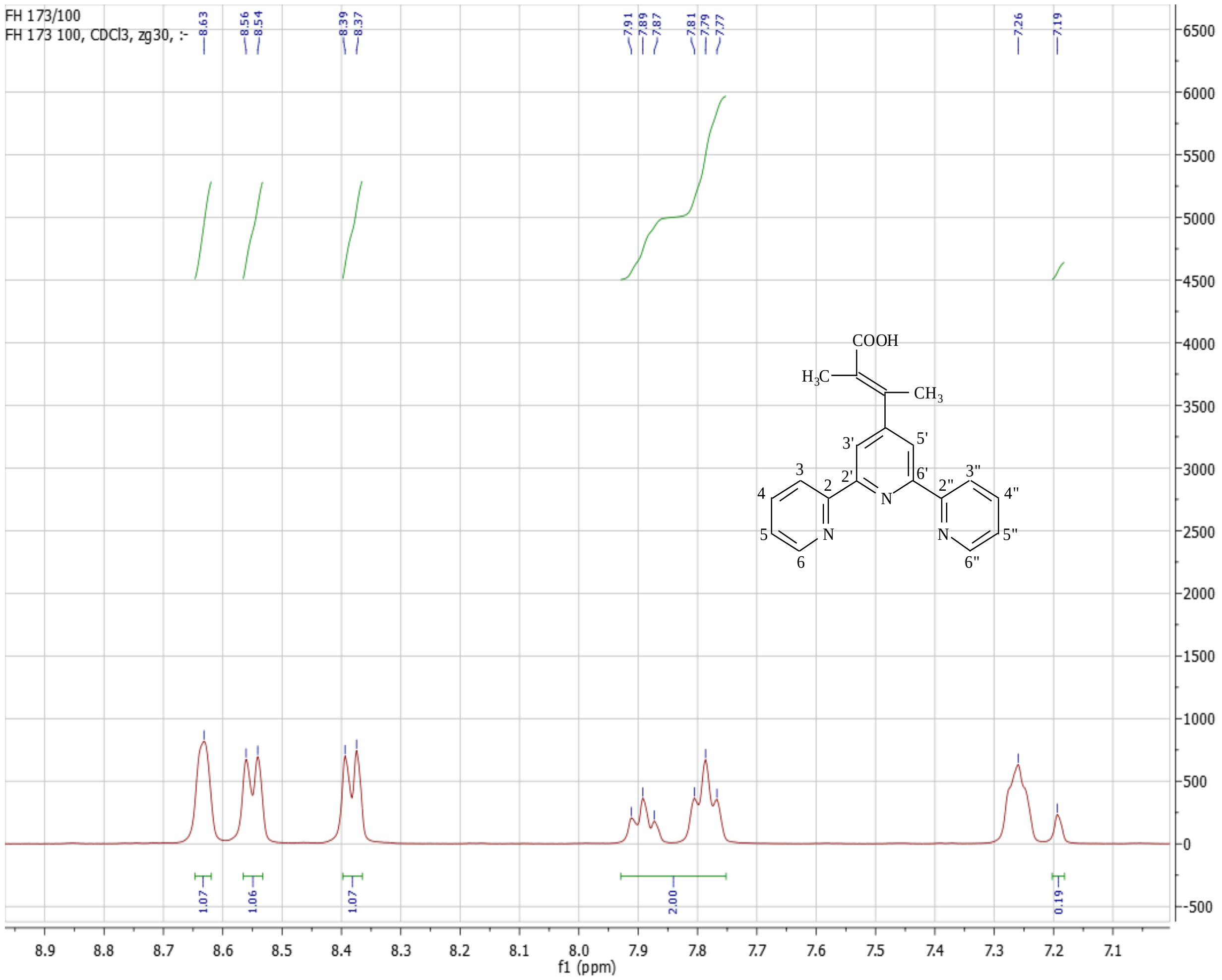

Supplementary 4. ^{1}H NMR spectrum of Complex [Ru(L1)$_2$(PF$_6$)$_2$].

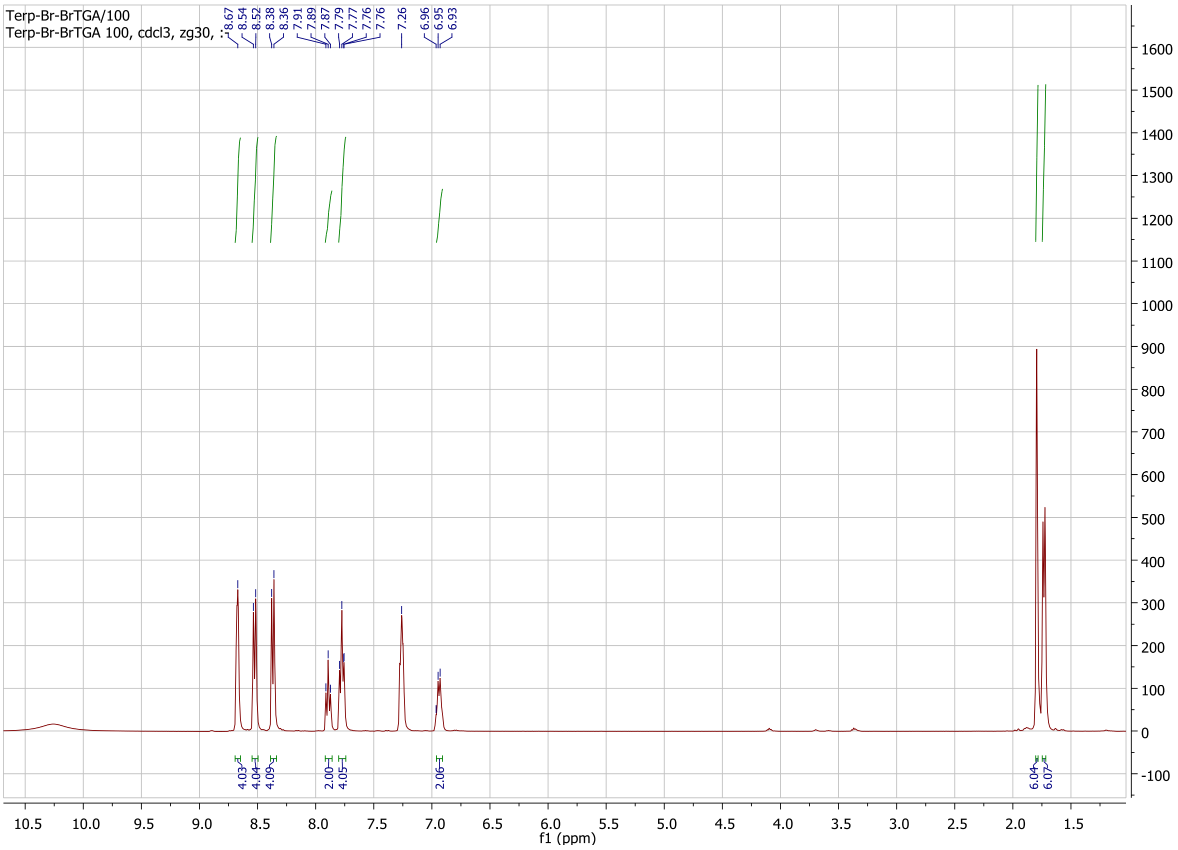

Supplementary 5. Aromatic region of 1H NMR spectrum of [Ru(L12)(PF6)2].

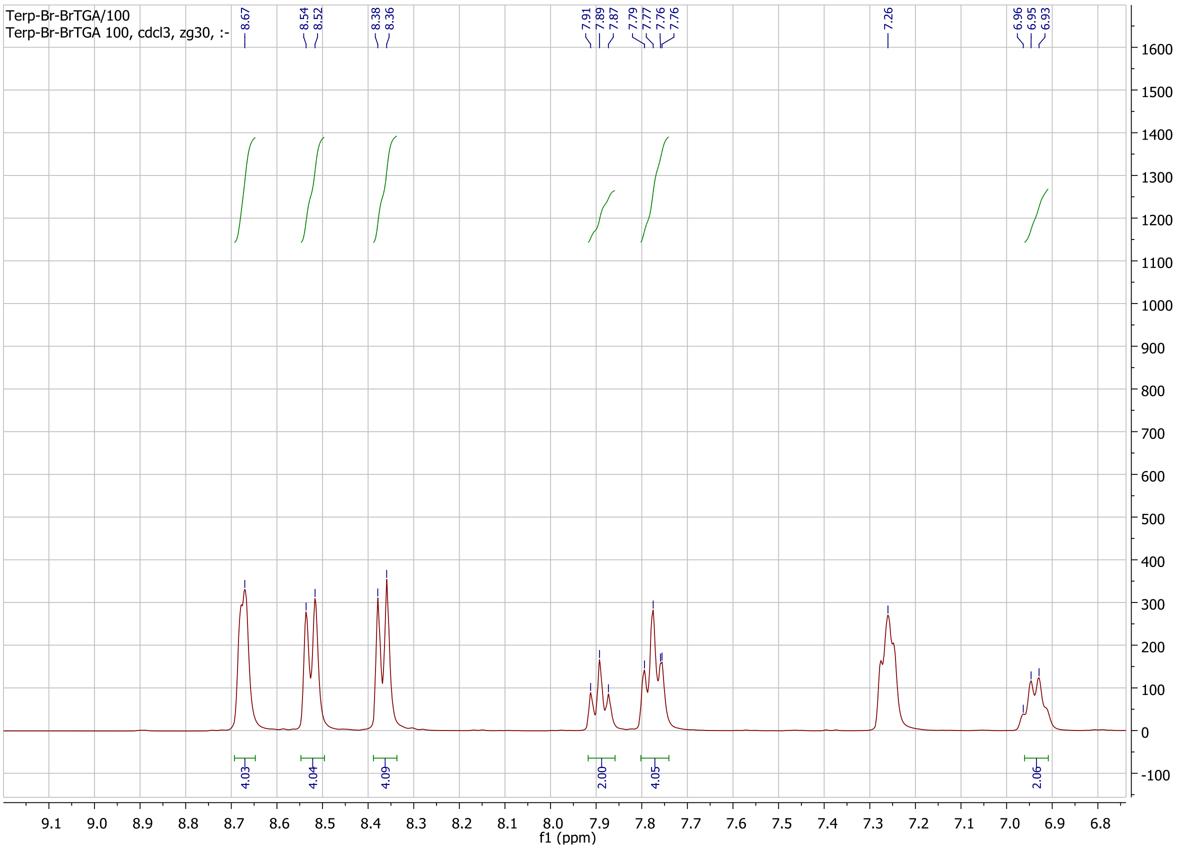

© 2012 by the authors; licensee MDPI, Basel, Switzerland. This article is an open access article distributed under the terms and conditions of the Creative Commons Attribution license (http://creativecommons.org/licenses/by/3.0/).
